# Some Remarks on Hernia

**Published:** 1908-12-19

**Authors:** 


### SURGERY.

### SOME REMARKS ON HERNIA.

### VII.—UMBILICAL HERNIA.

Cases of umbilical hernia may be divided into two classes—(*a*) congenital and (*b*) acquired. The congenital variety consists of a protrusion of intestine and omentum through an aperture where the umbilicus should be, whose presence is due to imperfect development of the muscles of the anterior abdominal wall. The extruded coils are contained in the umbilical cord, and if the hernia is a small one the condition may pass unrecognised and the gut may be included in the ligature by the midwife, and even cut across, with results which are necessarily disastrous to the patient. It is therefore highly important to appreciate the state of affairs when the child is born. When the presence of such a hernia is recognised surgical interference is demanded, since, if the condition is left untreated, peritonitis is more than likely to supervene when the cord separates. The operation itself is not difficult, and only differs from any other radical cure of an umbilical hernia in the extreme youth of the patient and in the presence of the unobliterated umbilical cord. It is best to make an elliptical incision which includes the attachment of the cord, dissect out the various structures, return the extruded coils and unite the two abdominal recti firmly.

The acquired variety may be again sub-divided into two classes according to the age of the patient (*a*) occurring in young children, and (*b*) in adults. In young children the hernial protrusion is usually through the navel itself, and is caused by the umbilical cicatrix giving way as the result of an increase in the intra-abdominal pressure. A common cause of the condition is whooping-cough.

These cases exhibit a remarkable tendency to undergo a spontaneous cure, and it is as certain as anything can be in surgery that the hernia will eventually disappear of its own accord, even if nothing be done for it. Efficient treatment will, however hasten the process. The best thing to do in an infant is merely to apply a firm binder round the abdomen in order to keep the coils back. If the pressure of the binder is not of itself sufficient, it may be reinforced by inserting in the front of the binder a flat pad of wood or metal. This should be considerably larger than the orifice in the abdominal wall. Under no circumstances should any projecting knob or button be used to occupy the hole; such treatment is most pernicious, and the only effect of it will be that it will tend to make the aperture permanent. For it stands to reason that it cannot close if its walls are constantly kept apart by a foreign body. It is only very occasionally that a case in a young child refuses to close. But if palliative treatment has been given a fair trial over a long period and the orifice shows no signs of closing a radical cure must be performed.

Umbilical hernia in adults is most common in elderly multiparæ. It is the outcome of a double cause (1) increase in the bulk of the abdominal contents, and (2) a weakening of the parietes, a condition often directly referable to repeated parturition. These herniæ may reach an enormous size, but the contents usually consist of omentum in great part; if any gut is present it is generally only one coil at the base of the hernia. In the early stages, when comparatively small, the hernia is reducible, but, as it increases in size, it becomes eventually a physical impossibility to return the extruded omentum into the abdominal cavity, because there literally is not room for it. In addition to this it is the rule for adhesions to form between the sac and its contents or between the contents themselves; this happens more constantly in umbilical than in any other form of hernia. The patient often complains of nothing else than the presence of an unsightly mass, but in other cases vomiting or some degree of intestinal obstruction may occur as the result of the traction of adhesions on the stomach or transverse colon.

The treatment of these cases is a matter of some difficulty. The ideal treatment is of course a radical cure; but this can only be really successful in a thin patient whose abdominal parietes are still moderately muscular. Unfortunately, as has already been mentioned, most are the exact antithesis of this. If, therefore, the hernia is reducible and does not give rise to symptoms, reliance should be placed on a well-fitting truss and an anti-fat diet; and even when the hernia is not completely reducible a hinge cup truss is often better than any operation.

But if one has reason to suspect the presence of adhesions, operative treatment is advisable, since the condition may go on to strangulation if left. The operation consists in making an incision over the hernia. It is usually covered with very thin skin which is redundant, and it is well, therefore, to make an elliptical incision and to remove a large portion of this. Adhesions are sought for and freed, and portions of the extruded omentum may be ligatured and removed. After this it may be possible to remove the sac and complete the radical cure by returning the rest to the abdomen and bringing the two recti together in front of it. But in some cases this is still impossible. For these an artificial device has been designed which has met with a considerable degree of success. It consists of a filigree of silver wire, about six inches long and four wide. This is placed between the parietal peritoneum and the muscles of the abdominal wall, and forms an artificial support. The recti are approximated as closely as possible in front of it. The method is comparatively recent, but those surgeons who have had experience of it have expressed themselves as being satisfied with their results. Theoretically, of course, it has the disadvantage that one is introducing a foreign body as a permanent measure.

Should an umbilical hernia become strangulated, the patient's condition is usually a desperate one. The strangulation may occur at the point of exit or be due to bands or holes in the omentum. In operating on such a case the whole hernia must therefore be thoroughly examined. It is not sufficient merely to reduce the hernia sac and all.

